# C-Peptide Prevents Hippocampal Apoptosis in Type 1 Diabetes

**DOI:** 10.1080/15604280214936

**Published:** 2002

**Authors:** Zhen-guo Li, Weixian Zhang, Anders A. F. Sima

**Affiliations:** 1 Department of Pathology Wayne State University School of Medicine Detroit Michigan USA; 2 Department of Neurology Wayne State University School of Medicine Detroit Michigan MI 48201 USA; 3 Morris J. Hood Jr. Comprehensive Diabetes Center Wayne State University School of Medicine Detroit Michigan USA; 4 Department of Pathology Wayne State University School of Medicine Room 9275 H.G. Scott Hall, 540 East Canfield Avenue 48201 Detroit Michigan USA

## Abstract

To explore mechanisms underlying central nervous system (CNS) complications in diabetes, we examined hippocampal neuronal apoptosis and loss, and the effect of C-peptide replacement in type 1 diabetic BB/W rats. Apoptosis was demonstrated after 8 months of diabetes, by DNA fragmentation, increased number of apoptotic cells, and an elevated ratio of Bax/Bcl-x_L_, accompanied by reduced neuronal density in the hippocampus. No apoptotic activity was detected and neuronal density was unchanged in 2-month diabetic hippocampus, whereas insulin-like growth factor (IGF) activities were impaired. In type 1 diabetic BB/W rats replaced with C-peptide, no TdT-mediated dUTP nick-end labeling (TUNEL)- positive cells were shown and DNA laddering was not evident in hippocampus at either 2 or 8 months. C-peptide administration prevented the preceding perturbation of IGF expression and reduced the elevated ratio of Bax/Bcl-x_L_. Our data suggest that type 1 diabetes causes a duration-dependent programmed cell death of the hippocampus, which is partially prevented by C-peptide.


*Int. Jnl. Experimental Diab. Res.*, 3:241–245, 2002
Copyright © 2002 Taylor & Francis
1560–4284/02 $12.00 + .00
DOI: 10.1080/15604280290013991

Taylor & Francis healthsciences

### C-peptide Prevents Hippocampal Apoptosis in Type 1 Diabetes

**Zhen-guo Li,[1,3] Weixian Zhang,[1,3] and Anders A. F. Sima[1,2,3]**

*Departments of [1]Pathology, [2]Neurology, and [3]Morris J. Hood Jr. Comprehensive Diabetes Center, Wayne State University School of Medicine, Detroit, Michigan, USA*

**To explore mechanisms underlying central nervous system (CNS) complications in diabetes, we examined hippocampal neuronal apoptosis and loss, and the effect of C-peptide replacement in type 1 diabetic BB/W rats. Apoptosis was demonstrated after 8 months of diabetes, by DNA fragmentation, increased number of apoptotic cells, and an elevated ratio of Bax/Bcl-$x_L$, accompanied by reduced neuronal density in the hippocampus. No apoptotic activity was detected and neuronal density was unchanged in 2-month diabetic hippocampus, whereas insulin-like growth factor (IGF) activities were impaired. In type 1 diabetic BB/W rats replaced with C-peptide, no TdT-mediated dUTP nick-end labeling (TUNEL)-positive cells were shown and DNA laddering was not evident in hippocampus at either 2 or 8 months. C-peptide administration prevented the preceding perturbation of IGF expression and reduced the elevated ratio of Bax/Bcl-$x_L$. Our data suggest that type 1 diabetes causes a duration-dependent programmed cell death of the hippocampus, which is partially prevented by C-peptide.**

**Keywords** Apoptosis; C-peptide; Diabetic BB/W Rats; Hippocampus

Clinical and experimental studies have suggested that type 1 diabetes may account for cognitive dysfunction in the absence of hypoglycemic episodes. A duration-dependent decline in cognitive function was reported in type 1 diabetic patients, who never experienced hypoglycemic episodes [1], and impaired intellectual and cognitive development has been demonstrated in children with type 1 diabetes [2]. Experimentally, spatial learning deficits in streptozotocin (STZ)-diabetic rats have been associated with altered synaptic integrity of hippocampus, findings which were modified by low doses of insulin [3]. We recently demonstrated a duration-related asynchronous apoptosis of hippocampal neurons in the spontaneously type 1 diabetic BB/W rat [4], which resulted in a 34% loss of $CA_1$ neurons after 8 months of diabetes. These changes were preceded and accompanied by a significant down-regulation of the hippocampal insulin-like growth factor (IGF) system consisting of IGF-1, IGF-2, IGF-1 receptor (IGF-IR), and insulin receptor (IR) [4, 5]. Because both IGF-1 and insulin exert antiapoptotic effects [6–9], we suggested that their decreased expression in type 1 diabetic hippocampus may underlie spatial learning deficits, apoptosis, and neuronal loss in the BB/W rats [4]. Earlier studies have shown that the insulinomimetic effect of C-peptide [10] prevented the abnormalities of IGF-1, IGF-IR, and IR expressions in peripheral nerve of the same type 1 diabetic animal model [11, 12], resulting in prevention of early metabolic as well as chronic structural changes characterizing type 1 diabetic polyneuropathy [13]. We, therefore, reasoned that C-peptide replacement of type 1 diabetic BB/W rats could potentially prevent the early abnormalities in the expression of the IGF system [5] in the central nervous system (CNS) and prevent subsequent hippocampal apoptosis and neuronal loss.

Received 4 June 2002; accepted 1 August 2002.
These studies were supported by grants from the Juvenile Diabetes Research Foundation (AAFS), the Thomas Foundation (AAFS), and the Wayne State University Morris Hood Jr. Diabetes Center (ZGL).
Address correspondence to Anders A. F. Sima, MD, PhD, Department of Pathology, Wayne State University, Room 9275, H.G. Scott Hall, 540 East Canfield Avenue, Detroit, MI 48201, USA. E-mail: asima@med.wayne.edu

### RESULTS AND COMMENTS

Prediabetic (n = 34) and non–diabetes-prone (n = 17) male BB/W rats were obtained from Biomedical Research Models (Rutland, MA). The animals were cared for in accordance with institutional and National Institute of Health (NIH) guidelines

(publication no. 85-23, 1995) and monitored as previously described [4, 13]. Following detection at 72 ± 4 days, all diabetic rats were treated with daily doses of protamine zinc insulin (Blue Ridge Pharmaceuticals, Greensboro, NC) to maintain blood glucose levels at 20 mmol/L. Half of the diabetic animals (n = 17) were replaced with rat-II C-peptide (75 nmol/kg/day; >98% purity by high-performance liquid chromatography [HPLC]; Genosys, Cambridge, UK) delivered via Alzet osmotic pumps (ALZA Corporation, Palo Alto, CA) from onset of diabetes. The other half of diabetic rats were sham-operated.

At 2 and 8 months of diabetes, both diabetic and C-peptide–replaced diabetic rats showed significant weight loss (both $P < .001$ versus control rats) and significantly elevated blood glucose levels (both $P < .001$ versus control rats) (Table 1). In C-peptide–replaced diabetic rats, serum C-peptide levels were normalized to 75% in 2-month and to 77% in 8-month diabetic rats (Table 1). The insulin doses required to maintain the desired hyperglycemic levels did not differ between diabetic and C-peptide–replaced diabetic rats (Table 1).

Total RNA was isolated from hippocampus, frontal cortex, diencephalon, and cerebellum by the acid guanidinium thiocynate–phenol–chloroform method [14]. The Northern blot transfer and hybridization were performed as described previously [4]. The mRNA expressions of IGF-1, IGF-2, IGF-IR, and IR in the hippocampus of 2-month diabetic rats was reduced to 50.1% ± 12.6%, 51.0% ± 10.7%, 53.4% ± 10.9%, and 54.4% ± 10.1%, respectively, of control values (all $P < .01$). C-peptide replacement partially prevented the decrease in expression of these genes to 73.3% ± 4.9%, 71.9% ± 5.8%, 76.0% ± 8.9%, and 73.2% ± 6.7%, respectively, of control values ($P < .01$ for all versus control rats, $P < .05$ for all versus diabetic rats) (Figure 1). In peripheral nerve, IGF-1 expression is decreased, whereas IGF-IR and IR are both increased in the BB/W rats. Interestingly, although these abnormalities differ from those in CNS, they are prevented by C-peptide replacement [11, 12]. These findings suggest that C-peptide, probably via its insulinomimetic effect [10, 15], modulates the expression of the IGF system both in the CNS and the peripherial nervous system (PNS), via as of yet unknown factors.

For demonstration of apoptosis, genomic DNA was extracted according to Ausbel and coworkers [16]. Nucleosomal DNA ladder was detected by ligand-mediated polymerase chain reaction (LM-PCR) method following the manufacturer's instruction (Clontech, Palo Alto, CA). For amplification of internal control, we used a primer set for glyceraldehyde-3-phosphate dehydrogenase (GAPDH) cDNA: 5′-ACCACAGTCCATGCCATCAC and 5′-TCCACCACCCTGTTGCTGTA [4]. NeuroTACS II kits (Trevigen, Gaithersburg, MD) were used for TdT-mediated dUTP nick-end labeling (TUNEL) assays on 6-$\mu$m paraffin sections [4]. TUNEL-positive neurons were expressed as a percentage of total neurons per hippocampal region ($CA_1$ to $CA_4$). Immunoblotting was performed as previously described [15]. Rabbit anti-Bax and anti–Bcl-$x_L$ antibodies and horseradish peroxidase (HRP)-conjugated secondary antibody were purchased from Santa Cruz Biotechnology (Santa Cruz, CA). The enhanced chemiluminescence (ECL) detection system was from Amersham Pharmacia Biotech (Piscataway, NJ).

At 2 months of diabetes, none of the animal groups showed evidence of hippocampal apoptosis, either by LM-PCR DNA laddering, TUNEL stain, or as indicated by Bax and Bcl-$x_L$ (data not shown). In 8-month non–C-peptide–replaced diabetic rats, LM-PCR showed DNA laddering in hippocampus

**TABLE 1**
Clinical data from 2- and 8-month diabetic and C-peptide–replaced BB/W rats and age-matched control rats.

| | Body weight (g) | Blood glucose (mmol/L) | Insulin dose (IU/day) | Serum C-peptide concentration (pmol/L) |
|---|---|---|---|---|
| 2-month control (n = 10) | 381 ± 24 | 4.9 ± 0.2 | — | 948 ± 146 |
| 2-month diabetic (n = 10) | 334 ± 19* | 20.1 ± 2.1* | 2.8 ± 0.5 | 23 ± 19* |
| 2-month diabetic + C-peptide (n = 10) | 341 ± 18* | 19.7 ± 2.4* | 2.7 ± 0.3 | 710 ± 52*,‡ |
| 8-month control (n = 7) | 492 ± 31 | 5.0 ± 0.2 | — | 997 ± 102 |
| 8-month diabetic (n = 7) | 357 ± 29* | 20.7 ± 2.4* | 2.3 ± 0.4 | 19 ± 15* |
| 8-month diabetic + C-peptide (n = 7) | 359 ± 23* | 20.9 ± 1.7* | 1.9 ± 0.3 | 771 ± 27*,‡ |

*$P < .001$ versus age-matched control rats. ‡$P < .001$ versus duration-matched untreated diabetic rats.

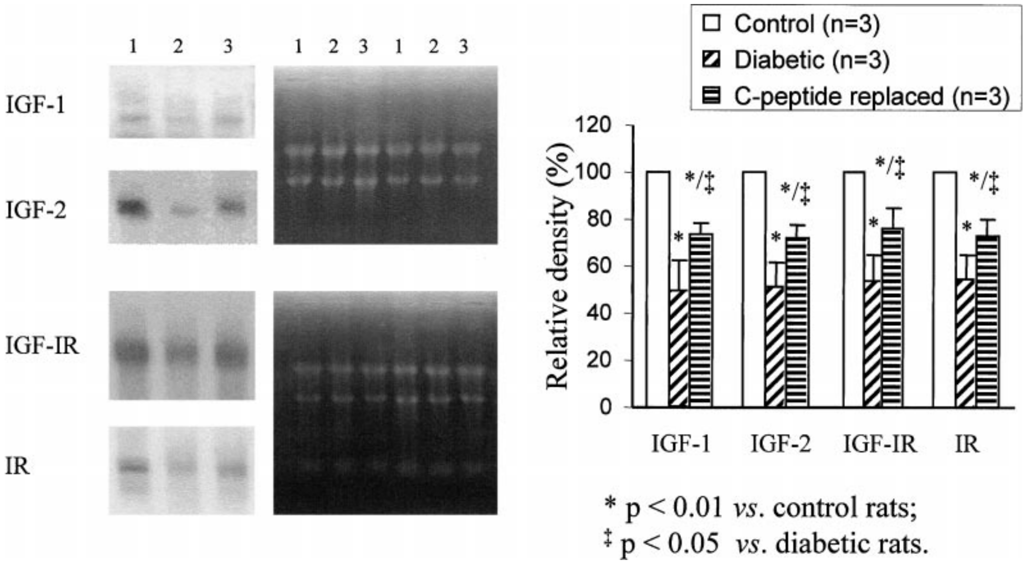

**FIGURE 1**
Northern blot hybridization. The mRNA levels of IGF-I, IGF-II, IGF-IR, and IR were reduced in hippocampus of 2-month diabetic BB/W rat and were partially reversed with C-peptide replacement (*left panels*). Ethidium bromide staining of the corresponding gel showed that approximately equal amounts of RNA were loaded into each lane (*middle panels*). Quantitation of mRNA expression (mean ± SD) in 3 separate experiments (*right panels*). Lane 1, control; lane 2, diabetic; lane 3, C-peptide–replaced diabetic.

and frontal cortex (Figure 2), which was accompanied by an increased percentage of TUNEL-positive hippocampal neurons (3.9% ± 1.0%; $P < .001$ versus control). The corresponding indices in control and C-peptide–replaced animals were zero. In 8-month diabetic rats, LM-PCR of DNA fragmentation was substantially prevented by C-peptide replacement in the hippocampus and fully prevented in frontal cortex (Figure 2).

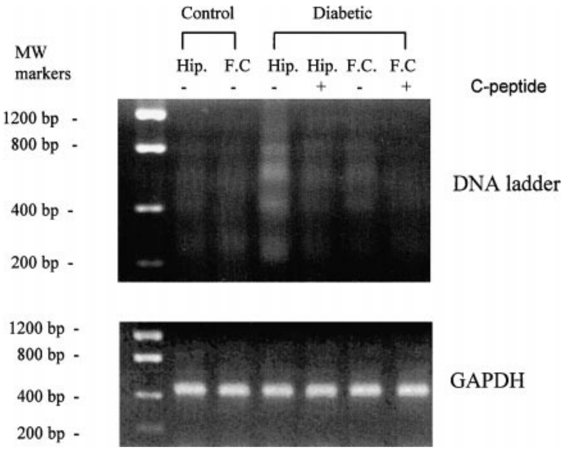

**FIGURE 2**
LM-PCR assay showing the effect of C-peptide on DNA fragmentation in 8-month diabetic BB/W rats (representative of 3 separate assays). Hip, hippocampus; F.C., Frontal cortex. DNA ladder was evident in hippocampus and frontal cortex in 8-month diabetic rats. It was faint in hippocampus and undetectable in frontal cortex in C-peptide replaced rats. Equal amounts of GAPDH genomic DNA were amplified as shown in the lower panel.

For neuronal density assessment, serial hemotoxylin-eosin–stained 6-$\mu$m-thick paraffin sections of hippocampus were used. They were analyzed using an Olympus BH-2 microscope and Image-Pro Plus 3.0 software (Media Cybemetics, Silver Spring, MD) [4]. The previously described asynchronous apoptosis, particularly affecting $CA_1$ [4], resulted in diabetic rats a 34.1% ± 4.3% loss of neurons in $CA_1$ ($P < .001$ versus control) and 24.1% ± 6.7% loss in $CA_2$ ($P < .05$ versus control) at 8 months of diabetes. C-peptide–replaced animals showed a partial prevention of hippocampal neuronal loss to 16.1% ± 5.2% in $CA_1$ ($P < .05$ versus diabetic rats) and to 12.3% ± 2.7% in $CA_2$ (nonsignificant versus control rats) (Figure 3).

These findings were associated with changes in apoptosis-related proteins in the hippocampus. The apoptosis-facilitating protein Bax was significantly increased in 8-month diabetic BB/W rat ($P < .01$ versus control), whereas the apoptosis-protecting protein Bcl-$x_L$ was unchanged, resulting in a 2.4-fold ($P < .01$) increase in the Bax/Bcl-$x_L$ ratio as compared to non-diabetic control rats. C-peptide replacement of diabetic BB/W rats significantly ($P < .05$) reduced the Bax expression, with no effect on Bcl-$x_L$, resulting in a 42% ($P < .05$) reduction of the Bax/Bcl-$x_L$ ratio compared to non–C-peptide–replaced diabetic rats (Figure 4).

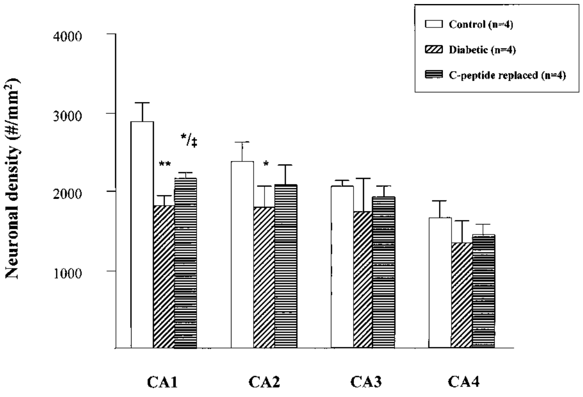

**FIGURE 3**
Effect of C-peptide on neuronal density in diabetic BB/W rats. Neuronal density was measured from serial 6-$\mu$m hemotoxylin-eosin–stained paraffin sections. The various hippocampal regions ($CA_1$ to $CA_4$) were calculated separately. Each bar represents mean ± SD from 4 animals. $^*P < .05$; $^{**}P < .001$ versus control; $^{\ddagger}P < .05$ versus diabetic.

These findings are in keeping with earlier reports that IGF as well as insulin action provide antiapoptotic effects [6–9]. Interestingly, in Alzheimer's disease, in which apoptosis has been invoked as a potential mechanism for hippocampal neuronal loss [17], the expression of IGF-1, IGF-IR, and IR are markedly reduced [18–20]. Because C-peptide shows an insulinomimetic effect [10, 15] mediated via the IR rather than the IGF-IR [21], the present findings are in keeping with those of Biessels and colleagues [3], who demonstrated that insulin therapy corrects long-term potentiation of the hippocampal $CA_1$ region in STZ-diabetic rats. Apoptosis can be induced via several cellular mechanisms, which most likely is also true for diabetic hippocampal apoptosis. In human neuroblastoma cells, we have demonstrated a potentiating effect of C-peptide on activation of nuclear factor kappa B(NF-$\kappa$B) and $Bcl_2$ [22], two mechanisms that have been invoked in apoptosis [23, 24]. Hence, there are probably multiple apoptotic pathways that are activated under type 1 diabetic conditions, some of which may not be corrected by C-peptide. The present data would suggest that this is the case, because C-peptide replacement only partially, although significantly, protected against hippocampal programmed cell death.

From these studies, we conclude that C-peptide replacement in type 1 diabetic BB/W rats has a protective effect on hippocampal apoptosis and neuronal loss. The duration-related occurrence of apoptosis is preceded by a down-regulation of the IGF system and IR, which is prevented by C-peptide replacement, suggesting that part of programmed neuronal cell death in type 1 diabetes may be mediated via impaired insulin and C-peptide actions.

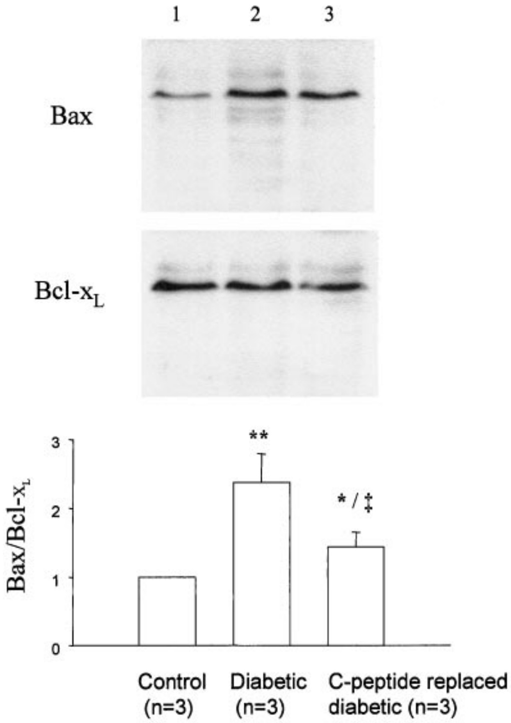

**FIGURE 4**
Western blot analysis of hippocampal Bax and Bcl-$x_L$ (representative of 3 blots). In diabetic animals (n = 3), there was an increased amount of Bax in the hippocampus, whereas Bcl-$x_L$ was unchanged, resulting in an increased Bax/Bcl-$x_L$ ratio ($P < .01$ versus control rats [n = 3]). C-peptide–replaced diabetic BB/W rats (n = 3) showed partial prevention of the increase in Bax, resulting in a significantly ($P < .05$ versus diabetic) lower Bax/Bcl-$x_L$ ratio. $^*P < .05$; $^{**}P < .01$ versus controls; $^{\ddagger}P < .05$ versus untreated diabetic animals. Lane 1, control rats; lane 2, diabetic BB/W rats; lane 3, C-peptide–replaced diabetic rats.

### REFERENCES

[1] Kramer, L., Fasching, P., Madl, C., Schneider, B., Damjancic, P., Waldhäusl, W., Irsigler, K., and Grimm, G. (1998) Previous episodes of hypoglycemic coma are not associated with permanent cognitive brain dysfunction in IDDM patients on intensive insulin treatment. *Diabetes,* **47,** 1909–1914.

[2] Schoenle, E. J., Schoenle, D., Molinari, L., and Largo, R. H. (2002) Impaired intellectual development in children with type 1 diabetes: Association with $HbA_1c$, age at diagnosis and sex. *Diabetologia,* **45,** 108–114.

[3] Biessels, G. J., Kamal, A., Urban, I. J., Spruijt, B. M., Erkelens, D. W., and Gispen, W. H. (1998) Water maze learning and hippocampal synaptic plasticity in streptozotocin-diabetic rats: Effects of insulin treatment. *Brain Res.,* **800,** 125–135.

[4] Li, Z. G., Zhang, W., Grunberger, G., and Sima, A. A. F. (2002) Hippocampal neuronal apoptosis in type 1 diabetes. *Brain Res.,* **946,** 221–231.

[5] LeRoith, D. (1999) Insulin-like growth factor. *Horm. Metab. Res.*, **31,** 41–42.

[6] Lee-Kwon, W., Park, D., Baskar, P. V., Kole, S., and Bernier, M. (1998) Antiapoptotic signaling by the insulin receptor in Chinese hamster ovary cells. *Biochemistry,* **37,** 15747–15757.

[7] Bertrand, F., Atfi, A., Cadoret, A., L'Allemain, G., Robin, H., Lascols, O., Capeau, J., and Cherqui, G. (1998) A role for nuclear factor $\kappa$B in the antiapoptotic function of insulin. *J. Biol. Chem.*, **273,** 3931–3938.

[8] Singleton, J. R., Randolph, A. E., and Feldman, E. L. (1996) Insulin-like growth factor I receptor prevents apoptosis and enhances neuroblastoma tumorigenesis. *Cancer Res.,* **56,** 4522–4529.

[9] Russell, J. W., and Feldman, E. L. (1999) Insulin-like growth factor-I prevents apoptosis in sympathetic neurons exposed to high glucose. *Horm. Metab. Res.,* **31,** 90–96.

[10] Grunberger, G., Qiang, X., Li, Z. G., Mathews, S. T., Shrissa, D., Shisheva, A., and Sima, A. A. F. (2001) Molecular basis for the insulinomimetic effects of C-peptide. *Diabetologia,* **44,** 1247–1257.

[11] Sugimoto, K., Zhang, W., Xu, G., Wahren, J., and Sima, A. A. F. (1998) Expression of insulin and IGF-1 receptors' mRNAs in peripheral nerve of type 1 diabetic BB/W-rat: Effect of C-peptide. *Diabetes,* **47,** A297 (Abstract).

[12] Sima, A. A. F., Zhang, W., Murakawa, Y., Wahren, J., and Pierson, C. R. (2002) C-peptide corrects aberrations in immediate early gene responses and cytoskeletal protein expression in regenerating nerve in type 1 neuropathy. *Diabetes,* **51** (Suppl. 2), A198 (Abstract).

[13] Sima, A. A. F., Zhang, W., Sugimoto, K., Li, Z. G., Wahren, J., and Grunberger, G. (2001) C-peptide prevents and improves chronic type 1 neuropathy in the BB/W-rat. *Diabetologia,* **44,** 889–897.

[14] Chomczynski, P., and Sacchi, N. (1987) Single-step method of RNA isolation by acid guanidinium thiocyanate-phenol-chloroform extraction. *Anal. Biochem.,* **162,** 156–159.

[15] Li, Z. G., Qiang, X., Sima, A. A. F., and Grunberger, G. (2001) C-peptide attenuates protein tyrosine phosphatase activity and enhances glycogen synthesis in L6 myoblasts. *Biochem. Biophys. Res. Commun.,* **280,** 615–619.

[16] Ausbel, F. M., Brent, R., Kingston, R. E., Moore, D., Seidman, J. G., Smith, J. A., and Struhl, K. (1994) *Current Protocols in Molecular Biology*. New York, Greene Publishing Associates and John Wiley & Sons.

[17] Roth, K. A. (2001) Caspases, apoptosis, and Alzheimer disease: Causation, correlation, and confusion. *J. Neuropathol. Exp. Neurol.,* **60,** 829–838.

[18] Terry, B. M., Cannon, J. C., Guong, L., Wands, J. R., and de la Monte, S. M. (2001) Abnormalities in insulin, IGF-I, and corresponding receptor (R) expression in Alzheimer disease. *J. Neuropathol. Exp. Neurol.,* **60,** 546 (Abstract).

[19] Frölich, L., Blum-Degen, D., Bernstein, H. G., Engelsberger, S., Humrich, J., Laufer, S., Muschner, D., Thalheimer, A., Türk, A., Hoyer, S., Zochling, R., Boissl, K. W., Jellinger, K., and Riederer, P. (1998) Brain insulin and insulin receptors in aging and sporadic Alzheimer's disease. *J. Neural. Transm.,* **105,** 423–438.

[20] Hoyer, S. (2002) The brain insulin signal transduction system and sporadic (type II) Alzheimer disease: An update. *J. Neural. Transm.,* **109,** 341–360.

[21] Zhang, W., Li, Z. G., and Sima, A. A. F. (2001) The effect of C-peptide on cell proliferation of human neuroblastoma cell with and without insulin or IGF-1. *Diabetes,* **50** (Suppl. 2), A190 (Abstract).

[22] Zhang, W., Li, Z. G., and Sima, A. A. F. (2002) C-peptide potentiates the anti-apoptotic effect of insulin via activation of Bcl-2 and NF-$\kappa$B *Diabetes,* **51** (Suppl 2), A197 (Abstract).

[23] Kaltschmidt, B., Uherek, M., Wellmann, H., Volk, B., and Kaltschmidt, C. (1999) Inhibition of NF-$\kappa$ B potentiates amyloid beta-mediated neuronal apoptosis. *Proc. Natl. Acad. Sci. U. S. A.,* **96,** 9409–9414.

[24] Deveraux, Q. L., Schendel, S. L., and Reed, J. C. (2001) Anti-apoptotic proteins. The bcl-2 and inhibitor of apoptosis protein families. *Cardiol. Clin.,* **19,** 57–74.

